# Compliance With the Cauda Equina Pathway: Results of a Closed-Loop Audit

**DOI:** 10.7759/cureus.20843

**Published:** 2021-12-31

**Authors:** Emmanuel Ago, Ghulam Dastagir Faisal Mohammed, Saad Maqsood, Momin Mohaddis, Prakash Chandran

**Affiliations:** 1 Trauma and Orthopaedics, Warrington and Halton Teaching Hospital NHS Foundation, Warrington, GBR; 2 Neurological Surgery, Salford Royal NHS Foundation Trust, Manchester, GBR; 3 Orthopaedics, Warrington and Halton Teaching Hospital NHS Foundation, Warrington, GBR

**Keywords:** spinal surgery, cord compression, neurosurgery, back pain, cauda equina syndrome

## Abstract

Introduction

The British Association of Spine Surgeons (BASS) and Society of British Neurological Surgeons (SBNS) recommend urgent MRI imaging and operative intervention in patients with suspected cauda equina syndrome (CES). Due to the lack of a 24-hour MRI service and the centralisation of neurosurgery to large tertiary centres, there is a need for an evidence-based protocol for the referral of patients presenting with back pain, with red flags to specialist tertiary neurosurgical centres.

Methods

The standard operating procedure (SOP) at our local hospital outlines steps in the assessment, triage and onward referral of patients presenting with symptoms of acute CES.

A closed-loop audit cycle was performed; the first cycle was between September and December 2020 and the second was between January and April 2021. Recommendations made after the first cycle were actioned prior to re-audit.

Results

There was 100% compliance regarding discussions with neurosurgery following MRI and appropriate management following neurosurgical advice. There was a 21.1% increase in appropriate discussions with neurosurgery by the emergency department (ED), increased accurate documentation of red flags (5% anal tone and 21% perianal sensation). There was a 53% decrease in senior ED doctor referral to neurosurgery, although 100% referrals were discussed with an ED senior prior to referral, and a 20% decrease in compliance regarding neurosurgery plan documentation.

Conclusion

We were able to improve our compliance with several aspects of the SOP using simple measures. We could not improve one aspect of SOP, namely, a discussion with the specialist centre being performed by a senior doctor.

Since CES requires timely management and early scanning, we recommend a robust protocol at the admitting hospital. This paper presents the protocol at our hospital and the rationale behind it. We discuss what affects our compliance with the SOP and how simple interventions have helped us improve.

## Introduction

Cauda equina syndrome (CES), a complex syndrome consisting of back pain with associated neurological deficit, is a surgical emergency that requires timely management; failure to do so might result in permanent lower limb weakness, bladder dysfunction and bowel dysfunction [1]. The British Association of Spine Surgeons (BASS) and Society of British Neurological Surgeons (SBNS) advise intervention as soon as safely possible. Furthermore, they recommend urgent MRI imaging at the admitting hospital [2]. There is increasing litigation regarding the long-term sequelae, including disability, of cauda equina and the profound effect it can have on the quality of life of individuals who were otherwise fit and well [3]. In the UK, between 2013 and 2016, there were 131 claims relating to CES, with a value of approximately £68 million [4-5].

Spinal services are centralised at specialist referral centres; non-specialist centres attend to these patients and arrange urgent MRI scans followed by a discussion with the specialist centre. At non-specialist centres, it is not always possible to get an MRI within a given time frame due to several logistical issues.

Due to the above reasons, there is a need for a robust protocol to guide the initial management at the admitting hospital (non-specialist centre) and the referral of these complex cases to the spinal specialist centre. These protocols are specific to each region but are broadly guided by recent literature and national guidelines in coordination with local spine referral centres.

We are a non-specialist admitting centre for acute back pain. We have in place a Standard Operating Protocol (SOP) for managing local back pain with suspected CES. The patient presents directly to the emergency department (ED( or can be referred by a general practitioner to the trauma and orthopaedic on-call team. They are then assessed at the admitting centre and referred to the spine referral centre for advice. If there is no evidence of CES after discussion with the spine referral centre, the patient is transferred to the ward for pain management and therapy until they are safe to be discharged.

We audited our compliance against the suspected back pain and CES SOP, made interventions and closed the loop with a re-audit. We present the results of this closed-loop cycle and discuss the effects of simple interventions in increasing our compliance.

## Materials and methods

A closed-loop audit cycle against the suspected back pain and CES standard operating procedure (SOP) was performed. Data were provided by the Trust IT department on request, recorded on a Microsoft Excel spreadsheet (Microsoft Corporation, Redmond, WA) and stored on secure trust servers. Statistical analysis was via the Fisher Exacts test, performed on GraphPad Prism software (GraphPad Software Inc., California). This study was approved by the local clinical audit department. Ethical approval was not required, as data were collected for quality improvement and audit purposes; there was no direct involvement of human subjects.

**Figure 1:**
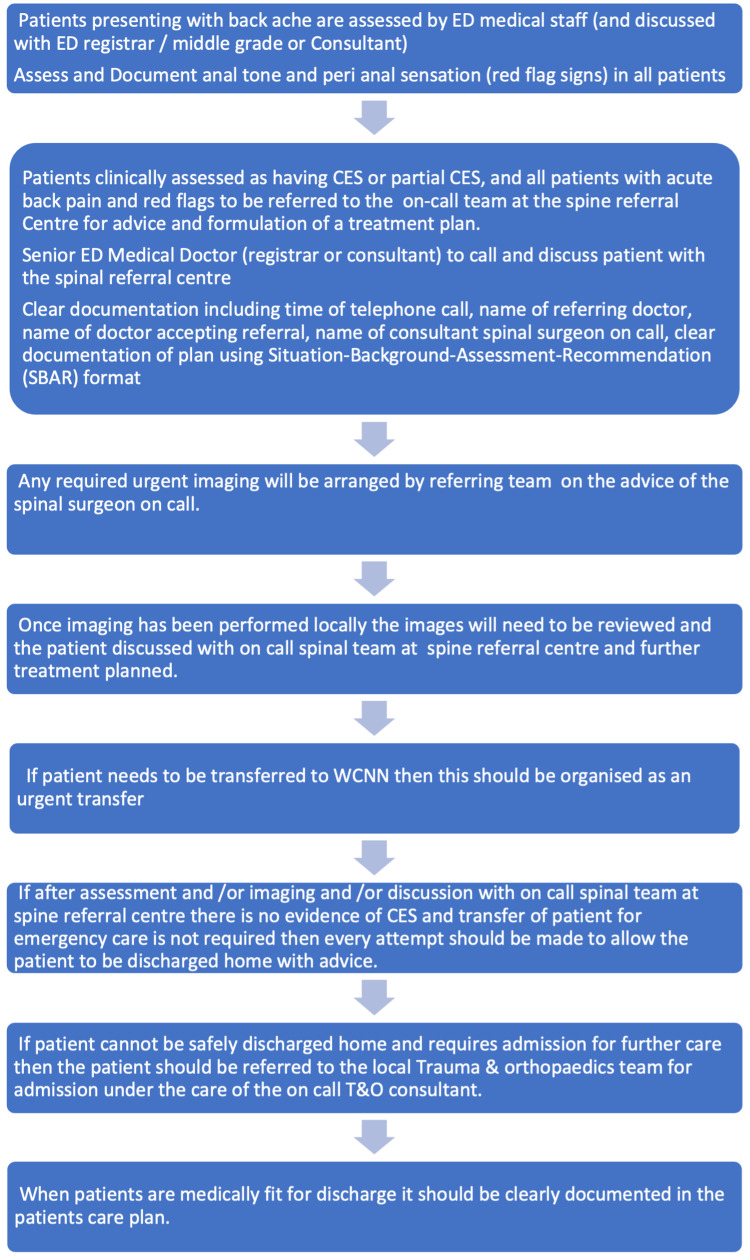
Standard Operating Procedure for Acute Back Pain With Suspected Cauda Equina Syndrome

A patient with back pain can present either directly to our emergency department or via referral from their general practitioner directly to orthopaedics. They are then assessed by ED doctors according to the protocol detailed in Figure 1. This protocol elaborately details the correct documentation, clinical signs to assess for and the need for an MRI scan within 24 hours. The information is relayed to a spinal referral centre via an online portal and via telephone as emphasised by the SOP. The spinal centre then advises on whether the patient can be managed locally or requires urgent transfer to the spinal centre. All locally managed patients are admitted under the care of trauma and orthopaedics.

We included adult patients presenting to our admitting non-specialist unit with symptoms of acute back pain and suspected CES. Our first cycle was between September 2020 and December 2020 and the second cycle was between January 2021 and April 2021. 

We audited our compliance against the following aspects of the SOP: initial assessment by ED staff and discussion with an ED senior doctor, discussion of patients with the spine referral centre, discussion between the spine referral centre and ED senior staff, assessment and documentation of anal tone, assessment and documentation of perianal sensation, documentation of the plan as per the SOP, MRI within 24 hours, re-discussion with the spine referral centre after MRI and appropriate management as per advice from neurosurgeons at the spine referral centre.

After the results of the first cycle, the following recommendations were actioned: 

1. Education: Presentation and teaching on CES and neurological examination to trauma and orthopaedic and emergency department junior doctors.

2. Dissemination of Information: Putting up posters to clearly display the pathway in the ED.

3. Discussion: Among trauma and orthopaedics and the ED to clarify the management of general practitioner referrals.

4. Inform: New and old staff were informed about the guidelines in the pathway via email.

## Results

**Figure 2:**
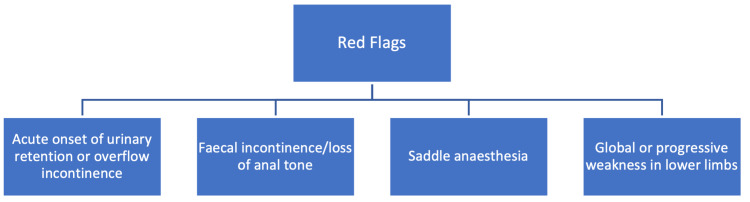
Red Flag Symptoms Outlined in the Cauda Equina Syndrome Standard Operating Procedure

The first cycle had 25 patients while the second cycle had 29 patients, with results and statistical significance presented in Table 1. There was 100% compliance demonstrated over the full audit cycle regarding four key aspects of the SOP. All patients identified as having red-flag symptoms (detailed in Figure 2) were discussed, as appropriate, with the spinal referral centre, and subsequently discussed following MRI imaging. Furthermore, all patients had MRI imaging within 24 hours of presentation to the ED and were treated in accordance with advice from the neurosurgeons at the referral centre.

**Table 1:** Results and Statistical Analysis of Compliance Over Two Cycles With Cauda Equina Syndrome Standard Operating Procedure (SOP)

	1^st^ Cycle N=25	2^nd^ Cycle N=29	Statistical Significance
Initial assessment by the ED team and discussion with a senior ED doctor	24 (96%)	26 (90%)	0.727
All patients with red flags discussed as appropriate with the spine referral centre	25 (100%)	29 (100%)	-
Discussion was done by an ED senior doctor	13 (61.9%)	2 (9%)	0.000
Assessment and documentation of anal tone in all patients	23 (92%)	28 (97%)	0.887
Assessment and documentation of perianal sensation in all patients	18 (72%)	28 (97%)	0.028
Spine referral centre plan documented in patient notes as per SOP	5 (20%)	0 (0%)	0.033
MRI within 24 hours	25 (100%)	29 (100%)	-
Discussion with the spine referral centre following MRI	25 (100%)	29 (100%)	-
Appropriate management as per the spine referral centre's advice	25 (100%)	29 (100%)	-

Compared with the first audit, there was a 21.1% (1st cycle 78.9% and 2nd cycle 100%, p=0.211, chi-square test) increase in appropriate discussions with the spine referral centre by the ED team and notable increases in accurate documentation of red flags on initial assessment, 5% and 25% for anal tone and perianal sensation, respectively.

There was a 53% decrease in senior ED doctors discussing with the spine referral centre and a 20% decrease in SOP compliance regarding spine referral centre plan documentation.

## Discussion

Cauda equina syndrome is a rare, as well as complex, syndrome with an annual incidence of approximately 1 in 100,000 [6-7]. Only 0.08% of patients with backache presenting to primary care and 0.27% presenting to secondary care had cauda equina and 19% of patients with clinically suspected cauda equina had cauda equina syndrome [4]. It is noted that all patients in both cycles were seen initially by junior ED doctors only. Delays occur due to junior doctors or nurses not being able to recognise CES [8]. Hence, discussion with a senior doctor is important and our compliance regarding this remained good over both cycles. It was found to decrease by 6% in the second cycle although this difference is statistically insignificant. We believe the Education component of our intervention helped in maintaining compliance.

Education and the dissemination of information via posters proved excellent in increasing our compliance in the examination and documentation of anal tone and perianal sensation. These two entities are not only of very high diagnostic value but also of prognostic value [9-10]. This improved the recognition of red-flag signs. Consequently, the inappropriate referrals to trauma and orthopaedics prior to referral to the spine centre decreased. We referred all patients with clinical partial CES or CES to the specialist spine centre in both cycles. Our interventions helped maintain this compliance as recognition of red flag signs improved.

Due to the complexities involved in assessment, investigation, management and communication, the SOP mandates that the telephone discussion with the spine centre be done by a senior doctor from the ED (registrar/middle grade/consultant), rather than junior doctors who may complete the online referral. Our compliance to this part of the SOP was lower than expected in the first cycle and worsened in the second cycle. We believe this is due to staff shortages and service pressures during the second cycle, which coincided with the coronavirus disease 2019 (COVID-19) pandemic. It could also be attributed to human factors; the referral is uploaded to an online portal and, as such doctors, might have felt they did not need to communicate with the on-call team at the specialist spinal centre. This is despite the SOP emphasising that patients be discussed via telephone, in addition to online referral, with the specialist to avoid any delays due to technical or logistical factors like the portal not being updated, not being viewed immediately by a specialist or the inability to add comments on the set portal proforma. Furthermore, junior doctors outnumber registrars and consultants in the ED and, as such, during busy periods, senior doctors would likely be occupied with emergencies, leaving junior doctors to make the referrals. Our interventions were not effective in improving compliance with this aspect and we believe further intervention in the form of a quality improvement project, with associated junior doctor surveys, is required to address this.

Due to medico-legal reasons, the SOP outlines documentation of the referral, including noting the time of discussion, name of the referring doctor, name of the doctor receiving the referral, name of the consultant on call at a referral centre and the discussion in SBAR (situation, background, assessment, recommendation) format. The names and timings were invariably present on the online referral system, as this is automated, but neither the online referral nor the documented notes had an SBAR format to show the complete extent of the discussion. The SBAR format has been shown to be effective in both communication and documentation [11]. During our teaching sessions, we emphasised the need for documentation using the SBAR format. We also created an online template on the electronic patient record system to be used for documentation. According to a systematic review, audits, feedback, education, electronic templates, and charts have been found to improve documentation standards in the ED [12].

Our compliance regarding MRI scanning within 24 hours, relaying this information to the spinal centre, and following their advice has been excellent through both cycles. The reason for compliance could be counterchecks by consultants, registrars, and junior doctors themselves, ensuring the plan has been followed and reflects the understanding of the seriousness of this syndrome. In addition, 100% compliance with time-dependent MRI scanning was maintained because of the prioritisation of these requests by the department of radiology.

## Conclusions

Cauda equina syndrome is a syndrome with significant implications for both patient and doctor. There is unequivocal evidence within the literature that early investigation and management of CES is imperative in ensuring the best treatment outcomes. Due to the lack of 24-hour MRI scanning services at many district general hospitals (DGHs) and the centralisation of neurosurgical services at tertiary specialist centres, robust, evidence-based protocols are required to facilitate the safe and expedient management of patients presenting with red flag symptoms to non-specialist centres.

This audit demonstrates that the implementation of an evidence-based SOP can improve the quality of care received by patients, and used in association with teaching and constant feedback, ensure the continued improvement of patient outcomes. Furthermore, it standardises management and quality of care provided to patients with CES and facilitates quantitative assessment of care, with scope for further simple interventions to increase compliance and subsequent quality of care. We believe this paper could be utilised as an example by other hospitals in creating evidence-based protocols for CES, auditing against their respective protocols and implementing simple measures to improve compliance.
